# Exponential history integration with diverse temporal scales in retrosplenial cortex supports hyperbolic behavior

**DOI:** 10.1126/sciadv.adj4897

**Published:** 2023-11-29

**Authors:** Bethanny P. Danskin, Ryoma Hattori, Yu E. Zhang, Zeljana Babic, Mikio Aoi, Takaki Komiyama

**Affiliations:** ^1^ Department of Neurobiology, University of California San Diego, La Jolla, CA, USA.; ^2^ Center for Neural Circuits and Behavior, University of California San Diego, La Jolla, CA, USA.; ^3^ Department of Neurosciences, University of California San Diego, La Jolla, CA, USA.; ^4^ Halıcıoğlu Data Science Institute, University of California San Diego, La Jolla, CA, USA.

## Abstract

Animals use past experience to guide future choices. The integration of experiences typically follows a hyperbolic, rather than exponential, decay pattern with a heavy tail for distant history. Hyperbolic integration affords sensitivity to both recent environmental dynamics and long-term trends. However, it is unknown how the brain implements hyperbolic integration. We found that mouse behavior in a foraging task showed hyperbolic decay of past experience, but the activity of cortical neurons showed exponential decay. We resolved this apparent mismatch by observing that cortical neurons encode history information with heterogeneous exponential time constants that vary across neurons. A model combining these diverse timescales recreated the heavy-tailed, hyperbolic history integration observed in behavior. In particular, the time constants of retrosplenial cortex (RSC) neurons best matched the behavior, and optogenetic inactivation of RSC uniquely reduced behavioral history dependence. These results indicate that behavior-relevant history information is maintained across multiple timescales in parallel and that RSC is a critical reservoir of information guiding decision-making.

## Teaser

Different neurons encode history across diverse timescales, which combine together to instruct the animal’s future decisions.

## INTRODUCTION

Integrating information from the past to make a decision is a universal and critical component of animal behavior. For instance, in value-based decision-making, animals establish a subjective value for each available action, based on the reward outcomes of actions taken in the recent past. Reinforcement learning (RL) models provide a simple but powerful framework for how to integrate historical information to guide future decisions (*1*). RL models such as the Rescorla-Wagner model (*2*) use the difference between expected rewards and observed rewards, known as reward prediction error (RPE), to update the subjective estimate of value. In typical formulations, the value associated with the action is updated by combining the new information from the most recent trial (reward prediction error, RPE) with the previous value estimates with a fixed learning rate. This update rule weighs the influence of recent outcomes more than outcomes in the distant past. Specifically, a fixed learning rate results in exponential decay in the influence of past outcomes, such that the influence decays with a fixed ratio for every unit of time. Exponential integration of the past is attractive because of its mechanistic simplicity: The brain would, in theory, only need to update its subjective value by combining, with a fixed rate, the ongoing value representation with RPE.

However, behavior studies across humans (*3*), nonhuman primates (*4*–*6*), and other animal models (*7*, *8*) engaged in value-based decision-making have observed that animal behavior deviates from exponential integration. Specifically, the integration of past experience generally exhibits a sharp initial drop on recent experience with a heavy tail on more distant experience, which is better fit by a hyperbolic than an exponential function. The adaptive advantage of such hyperbolic integration seems intuitive, as the difference in the environment between 1 and 2 min ago is likely more informative about the current environment than the difference between 1 month and 1 month plus a minute ago. Thus, it is beneficial to weigh the experience from 1 min ago more than 2 min ago but the weighting for a month ago and 1 month plus a minute ago should be nearly equivalent, which is achieved by heavy-tailed hyperbolic decay. Scaling the decay of information differentially across time imparts sensitivity to both recent changes and tendencies that are stable long term. However, the mechanism by which the brain performs hyperbolic-like integration of history is unknown.

To address this issue, we analyzed the history integration of cortical neurons in mice engaged in value-based decision-making. We find that behavioral integration of history in these mice is more hyperbolic than exponential, similar to previous behavioral studies. However, the history integration of individual cortical neurons is more exponential than hyperbolic. We provide a potential explanation for this apparent discrepancy between behavior and neurons by demonstrating that the time constants of exponential history integration are heterogeneous across neurons. Weighted averaging of these diverse exponential kernels, especially in the retrosplenial cortex (RSC) that overrepresents distant history information compared to other areas, can approximate hyperbolic-like behavioral integration. Inactivation of RSC, but not of the posterior parietal cortex (PPC) or posterior premotor cortex (pM2), impairs the use of history information. We propose that RSC neurons function as a pool of heterogeneous exponential history integrators, and appropriate weighting of these neural populations results in adaptive behavior with hyperbolic history integration.

## RESULTS

### History integration of mouse behavior is more hyperbolic than exponential

**Fig. 1.**
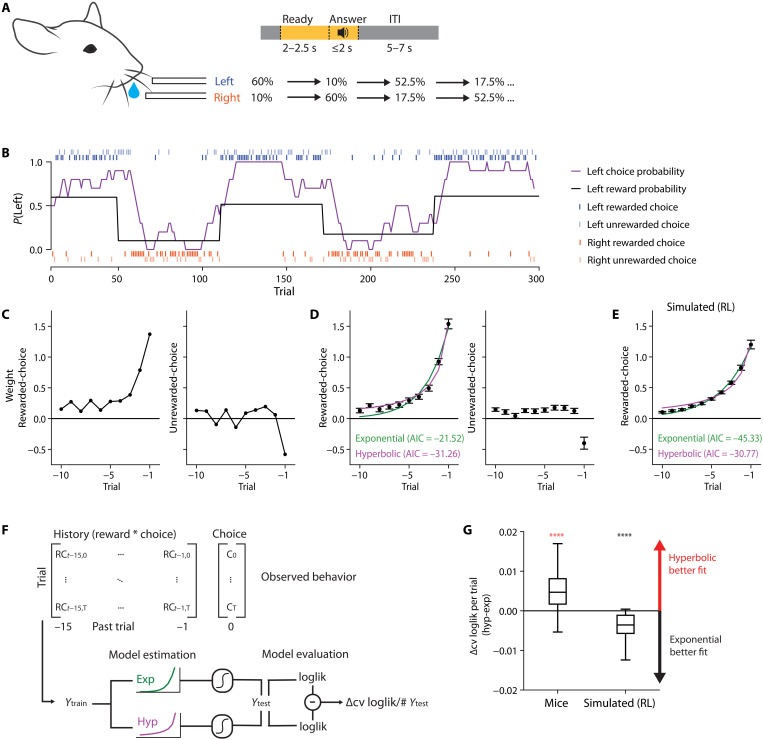
Mice rely on hyperbolic rather than exponential weighting of rewarded-choice history during history-dependent value-based decision-making. (**A**) Schematic of behavior task. Mouse was presented with two lick spouts with different probabilities of reward on the left or right side. The mouse was cued with an amber light-emitting diode to withhold licking during the ready period, and then cued with a tone to choose in the answer period. The reward contingency was inverted in a block structure of variable block lengths, and the pattern was repeated until the end of the session. The first block was randomly selected to be right or left high for any session. (**B**) Example session, probability of left reward assignment (black line), 10-trial smoothed choice pattern (purple line), left and right licks (blue, red) that were rewarded or unrewarded. (**C**) Logistic regression weights on rewarded-choice and unrewarded-choice history for the example session in (B). (**D**) Rewarded-choice and unrewarded-choice weights from logistic regression in black, grand mean across 74 sessions and 14 animals (means ± SEM). Exponential (green) and hyperbolic (magenta) curves fit to the mean rewarded-choice weights; exponential Akaike information criterion (AIC): −21.52, hyperbolic AIC: −36.26; lower AIC indicates better fit. (**E**) As in (D), but for 74 RL-simulated sessions, each with unique input parameters taken from mouse sessions; means + SEM. Exponential AIC: −45.33, hyperbolic AIC: −30.77; lower AIC indicates better fit. (**F**) Analysis workflow of the exponential and hyperbolic behavioral integration models. (**G**) Comparison of model performance, using 10-fold cross-validated log-likelihood, normalized by the number of trials, compared between exponential and hyperbolic models across identical train and test sets. Red indicates a median above zero, and black indicates a median below 0. Mice: *P* = 2.71 × 10^−5^, simulated: *P* = 4.96 × 10^−5^, linear mixed model, ****P < 0.0001.

To investigate the neural basis of history integration, we first analyzed the behavioral choice patterns of head-fixed mice trained on a dynamic foraging task. The behavioral data were originally presented in (*9*). In each trial, the mice were presented with the ready cue (light), followed 2 to 2.5 s later by the answer cue (tone), after which they chose one of two options: lick left or lick right. There was no cue that instructed mice to choose one over the other, but the two lickports had different probabilities of delivering a water reward (schematized in Fig. 1A). These probabilities were stable for periods of time but changed every 60 to 80 trials, without any cue to the mouse. Mice trained in this task dynamically adjusted their choice pattern according to their choice-outcome history (example session; Fig. 1B).

We quantified their use of the choice and outcome information from past trials with a logistic regression model. The model was fit using two types of history information: rewarded-choice history (the interaction between reward and choice: 1 for rewarded left choice, −1 for rewarded right choice, and 0 otherwise) and unrewarded-choice history (1 for unrewarded left choice, −1 for unrewarded right choice, and 0 otherwise) for recent trials. The regression weights for rewarded-choice history indicate that mice used the most recent history information more than distant history information, but with nonzero weights for trials as far as 10 trials back (Fig. 1, C and D). The shape of this decay exhibits a sharp initial drop on recent experience and a heavy tail on more distant experience, which is better described by a hyperbolic than exponential function [exponential Akaike information criterion (AIC): −21.52, hyperbolic AIC: −36.26; lower AIC indicates better fit]. That is, distant history is weighted more than expected from a consistent decay across all time steps. This is notable because a hyperbolic-like decay is a feature of the behavior that standard RL models are unable to capture. The unrewarded-choice history does not follow a smooth, monotonic decay, instead showing a negative weight on the most recent experience and positive weights on more distant history. In the rest of this study, we focus on the rewarded-choice history.

To confirm that the behavior described by the RL model exhibits exponential decay, we generated an artificial choice pattern in an emulation of our task using a modified form of the RL model developed previously for this behavior (*9*). The parameters of the RL model were taken from fitting the mouse behavior for each session. The sets of fitted parameters were then used with the generative model to produce simulated behavior, and the simulated choice patterns were fit with the same logistic regression model as above. By analyzing the history weights from the regression, we find that the simulated behavior is better fit by exponential than hyperbolic decay (Fig. 1E, exponential AIC: −45.33, hyperbolic AIC: −30.77), in contrast to the mouse behavior in Fig. 1D. Exponential behavior by the RL model is expected; the RL agent of the simulation uses a recursive style of integration that is time-invariant and therefore by definition exponential in nature. This result confirms that our analysis can accurately detect this feature.

The results so far, based on the history weights from regression fits, suggest that the mice use a hyperbolic-like integration rather than exponential integration to make their decision on a trial-by-trial basis. We tested this more directly by comparing the fits of two models where decay functions were convolved directly with the rewarded-choice pattern, rather than fit the regression weights post hoc (Fig. 1F). We constructed this model with the explicit constraint that past weights decay with either an exponential or hyperbolic decay function. We then assessed whether the model with an exponential or hyperbolic constraint better fit the observed behavior. Model fit was evaluated by the session-by-session difference in cross-validated (CV) log-likelihood of hyperbolic and exponential models, normalized by the number of trials. In this nomenclature, a log-likelihood difference larger than zero indicates that a hyperbolic constraint better fits the behavior, and less than zero that an exponential fits better. We observe that mouse behavior is better fit by the hyperbolic model (median = 4.69 × 10^−3^, *P* = 2.71 × 10^−5^, linear mixed model; see Materials and Methods and Fig. 1G). In contrast, the simulated behavior generated with the RL was better fit with exponential integration (median = −3.57 × 10^−3^, *P* = 4.96 × 10^−5^, linear mixed model; Fig. 1G), as expected. This difference was consistent across individual animals and was not sensitive to the range of past trials included in the exponential and hyperbolic models (fig. S1). These results establish that the mice are using a behavioral strategy that deviates from the standard RL model, integrating history information with a hyperbolic-like decay function.

### Cortical neurons encode rewarded-choice history with exponential integration

**Fig. 2.**
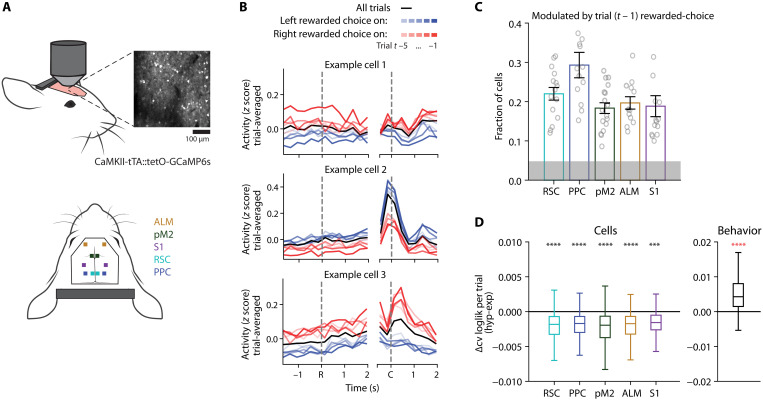
Cortical neurons encode history information with exponential decay. (**A**) Schematic of two-photon imaging from five cortical areas, showing one example field of view from RSC. One cortical area in one hemisphere was imaged in each session. (**B**) Trial-averaged activity of three example RSC neurons, aligned to the ready-cue onset (*R*), or the choice (*C*). The black line is the average across all trials. Blue lines are the mean of the subset of trials where the past trial (−5:−1 trials, indicated by darkening shade) was left choice and rewarded. Red lines are the same, but for the right choice and rewarded. (**C**) Fraction of cells significantly modulated by rewarded choice on the most recent trial (*t* − 1); means ± SEM. Gray shading indicates a fraction of significant cells in trial-shuffled data. *n* sessions: RSC = 15; PPC = 16; pM2 = 17; ALM = 12; S1 = 14. (**D**) Comparison of model performance, using 10-fold cross-validated log-likelihood normalized by the number of trials, between exponential and hyperbolic models across identical train- and test-sets. Left: Log-likelihood difference from the regression model fit to the cell activity; right: log-likelihood of the generalized linear model fit to the behavior. Behavior replicated from Fig. 1G for comparison. Note that the absolute values of log-likelihood for the cell model and behavior model cannot be directly compared because the magnitudes of the signals in the data are different. Linear mixed model, ***P < 0.001 and ****P < 0.0001.

To explore the neural basis of hyperbolic history integration, we analyzed neural activity recorded from task-performing mice. These data, originally described in (*9*), were acquired with in vivo two-photon calcium imaging in CaMKIIa-tTA::tetO-GCaMP6s double transgenic mice expressing GCaMP6s in cortical excitatory neurons (Fig. 2A). Fluorescence traces from each neuron were deconvolved (*10*, *11*) to give an approximation of underlying spiking activity. We focused our analysis on five cortical areas; RSC, PPC, pM2, anterior lateral motor cortex (ALM), and primary somatosensory cortex (S1).

The activity of a subset of cortical neurons was modulated by rewarded-choice history. As seen in three example cells imaged in the same session in RSC, shown in Fig. 2B, these cells exhibited different levels of activity depending on whether the left or right choice was rewarded in recent trials. The clearest separation in activity was when the most recent trial was rewarded on either the left side (darkest blue) or the right side (darkest red). Some of these cells showed stronger activity following left rewarded choice (e.g., cell 2) while others following right rewarded choice (e.g., cells 1 and 3). We focused the following analysis on the activity during the pre-choice, ready period (2 s after the ready cue onset), during which mice were withholding licking. We quantified the fraction of neurons modulated by rewarded choice on at least the most recent trial (*t* − 1; Fig. 2C), using linear regression (see Materials and Methods and Eq. 9). The fraction of significantly modulated neurons varied across sessions and across cortical areas but was always well above chance, as calculated by shuffling the neural activity across trials. Contra- and ipsi-preferring cells were mixed in both hemispheres and only pM2 showed a mild contralateral preference (fig. S2).

To investigate how these history-modulated neurons integrate history information, we applied the analogous model as for the behavior. Specifically, to each cell, we fit a pair of models in which past choice history was constrained to display either an exponential or hyperbolic decay and quantified the model’s prediction of cell activity with CV log-likelihood, normalized by the number of trials. In contrast to the mouse behavior, we found that the cell activity was generally better fit by the exponential integration model (Fig. 2D; see also Fig. 1G). The cells were more exponential than hyperbolic across all cortical areas we investigated (RSC: median = −1.82 × 10^−3^, *P* = 5.88 × 10^−7^; PPC: median = −1.70 × 10^−3^, *P* = 6.33 × 10^−8^; pM2: median = −1.95 × 10^−3^, *P* = 1.53 × 10^−8^; ALM: median = −1.73 × 10^−3^, *P* = 9.56 × 10^−6^; S1: median = −1.57 × 10^−3^, *P* = 6.25 × 10^−4^; behavior: median = 4.28 × 10^−3^, *P* = 2.71 × 10^−5^; linear mixed model; see also fig. S2).

### Cortical neurons encode temporal information with a wide variety of time constants

**Fig. 3.**
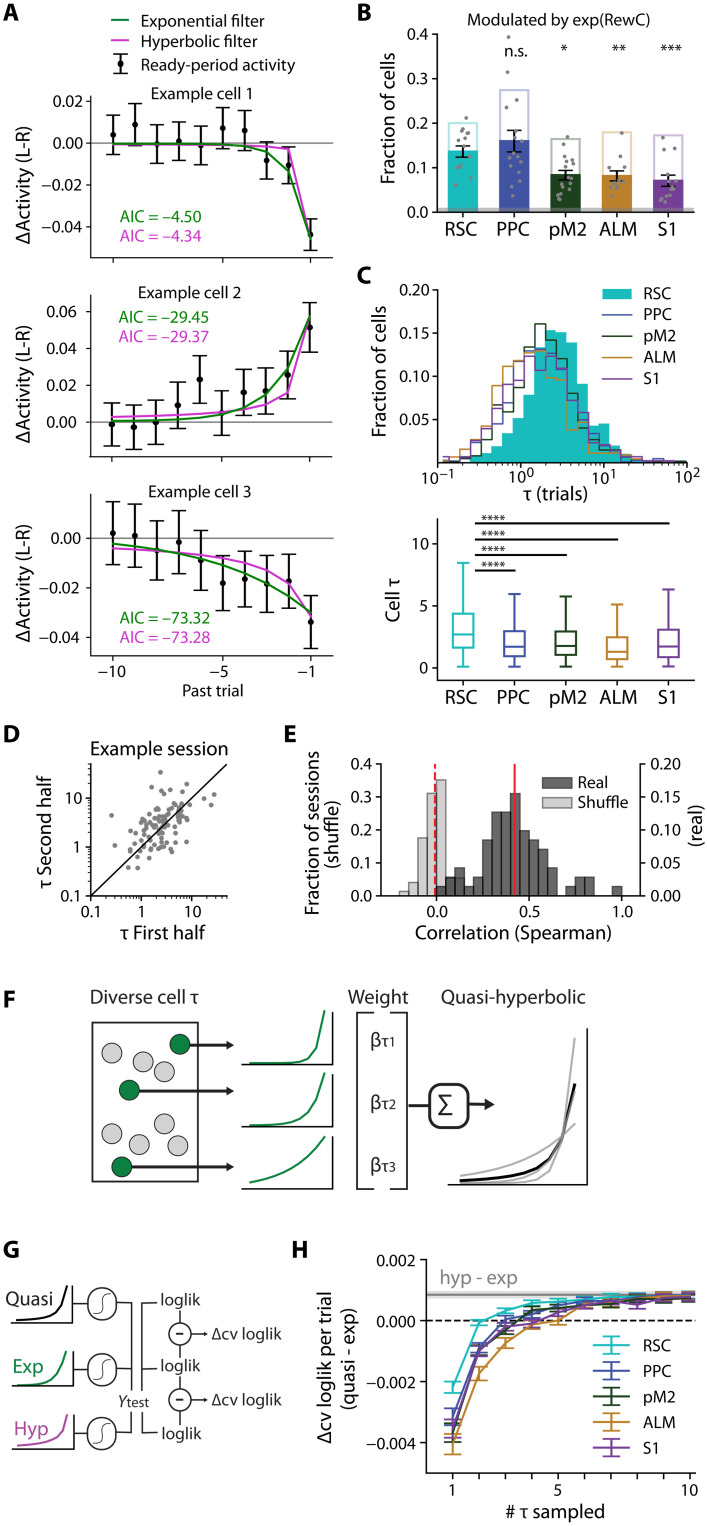
Neurons exponentially integrate with heterogeneous time constants, and the time constants in RSC cells best match the behavior. (**A**) Mean activity difference (left-right) from the 2-s “ready” period of Fig. 2B. Black dots are the difference between left rewarded activity and right rewarded activity (means ± SEM). Exponential filter (green line) estimated by the model for each cell. Cell 1: tau = 0.81; cell 2: tau = 1.45; cell 3: tau = 4.35. (**B**) Fraction of cells significantly modulated by rewarded-choice history with exponential decay in both halves of the session (filled bars, means ± SEM). Open bars are the fraction of cells modulated by at least the most recent trial (*t* − 1) reproduced from Fig. 2C. Gray shading is a fraction of cells significantly modulated in trial-shuffled data. Linear mixed model; *n* sessions: same as Fig. 2. (**C**) Distribution of exponential time constant τ across the significantly modulated cells in five cortical areas. Top: Histograms on a log axis. Bottom: Boxplots on a linear axis. All sessions for a given area are pooled. Bootstrapped test of medians compared to RSC, FDR-corrected for multiple comparisons. (**D**) τ Estimated separately in two halves of one example RSC session. Spearman’s *r* = 0.55, *P* = 9.30 × 10^−9^. (**E**) Distribution of the Spearman’s correlation across all session splits. All areas combined. Mean *r* = 0.42 (red line), geometric mean *P* = 2.39 × 10^−3^; shuffled data: mean *r* = −0.01 (dashed red line), geometric mean *P* = 0.3. (**F**) The quasi-hyperbolic model is the weighted sum of multiple exponential processes, yielding a heavy-tailed function approximating a hyperbolic. (**G**) Quantification of model performance by cross-validated log-likelihood. (**H**) Performance of the quasi-hyperbolic behavioral model based on a random sampling of time constants from each area, compared to the exponential behavioral model. Means ± SEM across 1000 random draws. The gray line and shading are means ± SEM of the hyperbolic model, reproduced from Fig. 1G. n.s., *P* > 0.05; *P < 0.05, **P < 0.01, ***P < 0.001, and ****P < 0.0001. n.s., not significant.

How can the brain generate behavior with hyperbolic integration when cortical neurons demonstrate exponential decay of past information? We consider the possibility that a hyperbolic discounting function with a sharp initial decay and a heavy tail can be approximated by a combination of exponentials with a variety of time constants. Therefore, if cortical neurons perform exponential integration with the decay time constants that are heterogeneous across neurons, then their combination could lead to a hyperbolic-like function to guide behavior. This mechanism for the generation of hyperbolic behavior from exponential neurons requires that there be a sufficiently diverse pool of neural decay rates to provide a basis for hyperbolic behavior. To test this idea, we examined the exponential decay time constants of history-modulated cells. We observed that even within one field of view for one cortical area, there are a wide variety of decay rates across cells (example cells from an RSC session; Fig. 3A). Take, for example, cell 1, which shows the sharpest convergence between the cell activity traces (Fig. 2B, top), corresponding to a short integration time (Fig. 3A, top). In contrast, example cell 3 still showed a clear separation of activity traces dependent on a rewarded choice as many as five trials back (Fig. 2B, bottom), leading to the more slowly decaying exponential fit in Fig. 3A (bottom). The mean activity difference (Figs. 2B and 3A) can be confounded by the autocorrelation of the behavior, such that adjacent trials tend to have similar choices and outcomes. Thus, to estimate the temporal decay of history information, we convolved the exponential decay function directly with the rewarded-choice history (see Materials and Methods, Eq. 10, and Fig. 3A, green line) rather than fitting a decay function on the mean activity difference.

To investigate the distribution of decay time constants across neuronal populations, we focused our analysis on cells that were significantly exponentially modulated by rewarded-choice history in both the first and second halves of the session. These stable, exponentially modulated cells represent a smaller fraction than the cells which are modulated at least by the most previously rewarded choice for some part of the session, but these fractions were well above chance from the shuffled distribution in all five areas (Fig. 3B). RSC and PPC exhibited the highest fraction of modulated cells, while pM2, ALM, and S2 all showed less prevalent encoding of rewarded-choice history information (compared to RSC by linear mixed model: PPC: *P* = 0.57; pM2: *P* = 0.03; ALM: *P* = 1.32 × 10^−3^; S1: *P* = 2.31 × 10^−4^).

We observed that these exponential cells show a wide variety in their decay time constants (Fig. 3C). The distributions of time constants differed across areas. RSC was particularly enriched in neurons that encode history information with longer time constants: There was a right shift in the distribution of tau in RSC compared to the other cortical areas (medians, RSC: 2.70; PPC: 1.71; pM2: 1.77; ALM: 1.30; S1: 1.72; compared to RSC, PPC: *P* < 1.0 × 10^−5^; pM2: *P* = 1.3 × 10^−5^; ALM: *P* < 1.0 × 10^−5^; S1: *P* = 2.0 × 10^−5^; bootstrapped test of medians, *P* values FDR-corrected for multiple comparisons; see also fig. S3).

To confirm that these distributions are not simply due to estimation noise, we partitioned each session into two nonoverlapping blocks of trials and evaluated the exponential time constants separately in each block for each neuron. As shown in the example RSC session in Fig. 3D, the time constants estimated in two halves of the session for the same neuron were consistent (Spearman’s *r* = 0.55, *P* = 9.30 × 10^−9^). Across cortical areas, we routinely found that cell-specific tau was consistent, with correlations substantially higher than trial-shuffled data (real data: mean *r* = 0.42, geometric-mean *P* = 2.39 × 10^−3^; shuffled data: mean *r* = −0.01, geometric-mean *P* = 0.39; Fig. 3E). This consistency indicates that each neuron integrates history with a time constant that is specific to the cell and consistent throughout a session and that this time constant can be reliably estimated.

Having established that multiple temporal scales are encoded simultaneously across neural populations in the cortex, next, we considered how the observed distributions of time constants, which differed across areas, could relate to the behavioral strategy. Specifically, we asked whether a weighted sum of these diverse exponential functions could approximate the hyperbolic-like integration observed in behavior. To answer this, we designed a linear regression model in which behavioral choice patterns of mice were fit by the weighted sum of multiple exponential integrators with different time constants. The time constants were randomly sampled from those observed in cortical neurons, and the weights associated with each of the time constants were fit to the behavior (schematized in Fig. 3F). We quantified the fit of this model with weighted sum of exponentials, which we call the “quasi-hyperbolic” model, as the difference in the CV log-likelihood from the performance of the model with a single exponential function with the best-fit time constant from the behavior (analysis outlined in Fig. 3G). We varied the number of time constants for the quasi-hyperbolic model, and for each number of time constants, the random sampling of time constants was repeated 1000 times and the results were averaged for each session. As we increased the number of sampled time constants, the performance of the quasi-hyperbolic model improved, surpassing that of the best-fit single exponential model and converging to the performance of the hyperbolic model (Fig. 3H, gray line being the average improvement of the hyperbolic model from Fig. 1G). The performance of the quasi-hyperbolic drawn from the RSC neurons improved most quickly with an increasing number of sampled time constants, indicating that RSC temporal encoding best matches the temporal characteristics of the behavior. Put another way, a downstream readout of information from RSC can reproduce the observed timescale of the behavior more parsimoniously than any other area. We note that, although a small number (<10) of time constants are sufficient for saturated performance in this analysis that assumes noiseless exponential integrators, the real neurons are noisy and thus would require a larger number of neurons. This suggests that RSC holds a unique position among these cortical areas as having a representation of temporal history information that best matches the temporal component of the behavior.

### Inactivating RSC, but not PPC or pM2, reduces the mouse’s use of rewarded-choice history in hyperbolic-like integration

In the above results, we laid out evidence that history integration occurs at multiple timescales simultaneously across different neurons in the cortex, and RSC is enriched in the timescales that best match the behavior. From this, we hypothesize that RSC is uniquely required for the history integration to guide the behavior.

**Fig. 4.**
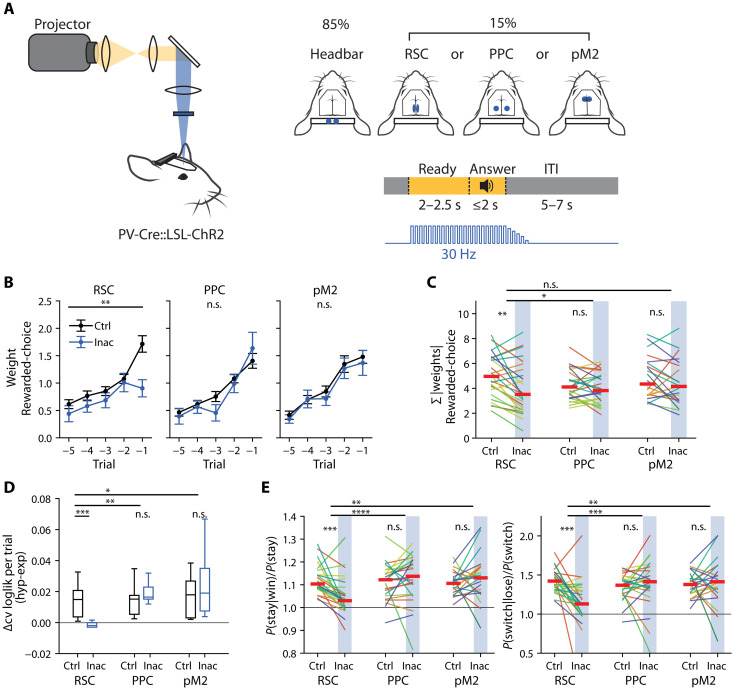
Inactivation of RSC reduces reliance on rewarded-choice history and impairs hyperbolic weighting of past trials. (**A**) Schematic of inactivation. Patterns of light delivered with a projector-based system onto the cortical surface of mice performing the task. Right: The position of stimulus for RSC, PPC, or pM2 during 15% of trials, one area per session, and to the headbar in the other 85% of trials. Illumination at 30 Hz during the ready and answer periods of all trials, with a linear ramp down of intensity over 100 ms (pulse widths not to scale). (**B**) Logistic regression weights in control (Ctrl, black line) and inactivation (Inac, blue line) trials. Means ± SEM. RSC: *n* = 10 mice, 26 inactivation sessions; PPC: *n* = 10 mice, 26 inactivation sessions; pM2: *n* = 9 mice, 22 inactivation sessions. Linear mixed model on the sum of the absolute rewarded-choice weights: RSC: *P* = 1.31 × 10^−3^; PPC: *P* = 0.27; pM2: *P* = 0.38. (**C**) Pairwise comparison of the inactivation effect on the sum of the absolute rewarded-choice history weights, Σ|weights|. Each line is one session, each color is a separate animal. Linear mixed model, within area repeated from (B). For across area, RSC-PPC: *P* = 0.04; RSC-pM2: *P* = 0.24. (**D**) Comparison of model performance by cross-validated log-likelihood. Model pairs trained separately on control or inactivation trials. RSC: *P* = 3.92 × 10^−4^; PPC: *P* = 0.31; pM2: *P* = 0.43; RSC-PPC: *P* = 5.65 × 10^−3^; RSC-M2: *P* = 0.02; linear mixed model. (**E**) Pairwise comparison of win-stay and lose-switch probabilities under inactivation. *P*(stay|win) is normalized by the overall stay probability [the average of *P*(stay|win) and *P*(stay|lose)]. *P*(switch|lose) was normalized by the overall switch probability [the average of *P*(switch|win) and *P*(switch|lose)]. Each line is one session, each color is a separate animal. Linear mixed model, n.s. *P* > 0.05; *P < 0.05, **P < 0.01, ***P < 0.001, and ****P < 0.0001.

To test this, we selectively and reversibly inactivated cortical areas via optogenetic activation of parvalbumin-positive (PV) inhibitory neurons in PV-Cre::LSL-ChR2 double transgenic animals that expressed channelrhodopsin-2 in PV neurons. We focused on RSC, PPC, and pM2. We used a projector system (*9*, *12*, *13*) to apply blue light over each cortical area. This flexible light delivery system, combined with a large cranial window preparation (*14*), allowed us to investigate the role of multiple cortical areas separately within the same animal (schematic in Fig. 4A). Inactivation was performed only for one area per session. In each session, inactivation occurred on a subset of trials (15% randomly selected, with the constraint to not be within three trials of each other), starting from the beginning of the ready cue until the choice was made. In all other trials, the light was directed over the headbar, away from the brain, in the same task period to control for light distraction. The total area of light coverage and intensity was consistent for each condition. Three of the 12 RSC inactivation animals have been previously described (*9*).

To quantify the effects of inactivation, we fit a modified version of the logistic regression analysis (see Materials and Methods and Eqs. 3 and 4) in which the inactivation trials had a separate set of weights from the control trials (see Materials and Methods). We found that inactivating RSC during the pre-choice, “ready” period reduced the dependence on rewarded-choice history (Fig 4, B and C). This effect was not seen with inactivation of PPC or pM2 (effect of inactivation condition: RSC: *P* = 1.31 × 10^−3^; PPC: *P* = 0.27; pM2: *P* = 0.38, linear mixed model on the sum of the absolute rewarded-choice weights; Fig. 4, B and C). Thus, of these three areas with a strong representation of history information, we found that RSC is uniquely necessary for the behavioral use of rewarded-choice history.

Next, we investigated whether inactivation affected the hyperbolic nature of behavioral history integration. The behavioral decay models, hyperbolic and exponential, were fit separately for the inactivation trials or control trials, and model performance was compared as the difference in CV log-likelihood between hyperbolic and exponential models. We found that RSC inactivation caused the behavioral strategy to become less hyperbolic than in the control condition. This reduction was not seen with inactivation of PPC or pM2 (RSC: *P* = 3.92 × 10^−4^; PPC: *P* = 0.31; pM2: *P* = 0.43, RSC-PPC: *P* = 5.65 × 10^−3^; RSC-M2: *P* = 0.02, linear mixed model; Fig. 4D). These results indicate that RSC is uniquely necessary for implementing the hyperbolic-like integration we observe in the choice patterns.

We additionally performed a separate analysis, quantifying the probability that the mouse would repeat the same action after a rewarded trial (“win-stay”) or switch after an unrewarded trial (“lose-switch”), normalized by its overall probability to either “stay” or “switch” (Fig. 4E). RSC inactivation was unique in reducing the probabilities of win-stay and lose-switch, which was not observed in inactivation of PPC and pM2 (win-stay, RSC-PPC: *P* = 7.38 × 10^−5^; RSC-pM2: *P* = 1.08 × 10^−3^; lose-switch, RSC-PPC: *P* = 5.23 × 10^−4^; RSC-pM2: *P* = 2.45 × 10^−3^, linear mixed model). This analysis, which does not rely on regression, confirms that RSC is critical for history-dependent adaptive decision-making.

## DISCUSSION

**Fig. 5.**
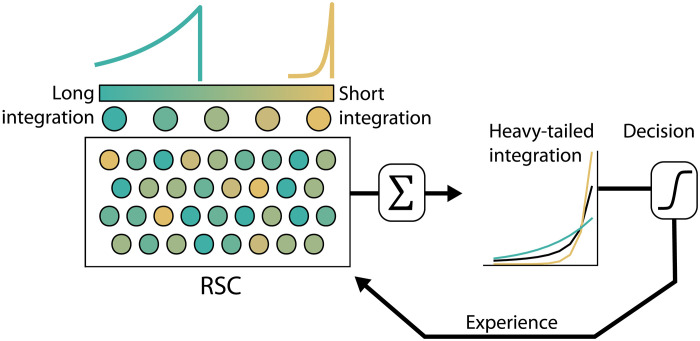
RSC as a reservoir of exponential history integrators with diverse integration windows used for decision-making. RSC neurons encode rewarded-choice history experience with a diversity of exponential time constants, including cells with short integration and long integration. The combination of many exponential integrators yields the heavy-tailed integration observed in the behavior.

Here, we showed that the behavior of mice engaged in value-based decision-making is driven by trial history integrated according to a hyperbolic-like decay, similar to what has been shown in analogous behavioral tasks in multiple species (*3*–*8*). In an apparent contradiction, we found that cortical neurons encode history in a manner more consistent with an exponential decay than hyperbolic. However, cortical neurons do not represent history homogeneously. Rather, history is encoded simultaneously across many neurons with heterogeneous time constants of integration. This activity pattern is consistent with a series of exponential processes acting in parallel over widely distributed temporal horizons, which can sum together to yield the heavy-tailed, hyperbolic-like integration observed in the behavioral strategy. RSC encodes this information over a longer temporal horizon than the other cortical areas, and the inactivation of RSC uniquely attenuates the use of history information and impairs the hyperbolic-like integration. From these results, we posit that history information is integrated in a distributed and diverse manner, with experience across different timescales encoded differently across neurons. We propose a model where history information is encoded in individual neurons by simple exponential integration with heterogeneous time constants. Behavior arises from the combination of multiple exponentially integrative processes, in particular those with longer time constants, which yields a decision strategy that has the heavy-tailed feature of a hyperbolic function (Fig. 5).

This model provides a potential mechanism by which the brain uses a conceptually simple mechanism of exponential value updates to achieve a hyperbolic-like behavior. Such a heavy-tailed integration has also been described as the phenomenon of “undermatching” and has previously been considered a suboptimal form of decision-making in laboratory behaviors (*5*, *6*, *8*). However, a natural environment has dynamics at multiple timescales. For example, the decision of when and where to go foraging for food may depend on factors such as the weather, hunger state, and seasonal changes in available items, just to name a few. These factors vary across orders of magnitude in the speed of changes, rendering an exponential integration with a constant decay rate per unit time to be suboptimal. Hyperbolic-like decay is conserved across species and may be an evolutionary response to an environment that has multiple timescales of changes. Although, here, we focused on retrospective processes in which historical information decays over time, prospective discounting of potential future rewards is also known to follow a hyperbolic function. For example, in delay discounting experiments in which the animal is presented with choices that return rewards at different temporal delays and magnitudes, animals show a stereotypic reversal in time preference at long lags indicative of hyperbolic discounting (*15*, *16*). Thus, hyperbolic functions seem common in weighting influences of events over time. However, truly hyperbolic computation is difficult to achieve with a recursive operation modeled in standard RL, and other computations that approximate hyperbolic discounting have been proposed (*17*–*20*). In contrast, exponential computation can be achieved with a simple recursive operation. In an extension of this logic, our model proposes that multiple exponential computations with heterogeneous time constants performed in parallel can generate hyperbolic-like behavior.

We interpret the heavy-tailed influence of history on behavior as a form of RL with hyperbolic-like integration of choice-outcome information. However, we wish to note that the heavy-tailed behavior can also be attributed to choice perseverance that is independent of the outcome. The current experiments do not allow us to dissociate the contributions of choice-outcome history and outcome-independent perseverance. This likely also contributes to the non-monotonic decay of unrewarded-choice weights. Regardless, a parsimonious observation is that history influences behavior with a heavier tail than expected from exponential RL, and we propose that this arises from individual neurons performing history integration with heterogeneous time constants.

The inactivation of RSC reduced, and did not eliminate, the behavioral reliance on the rewarded-choice history information, and this effect was the clearest for the most recent history. It is likely that long time-constant information, enriched in RSC, is redundantly encoded in areas not examined in the current study. Distributed encoding would make distant history information more resistant to the short-term perturbation that we used. Previous studies have also described heterogeneity across individual neurons in their encoding of behavior-related temporal information within and across brain areas (*21*–*27*). We extend these observations and uncover that RSC is especially enriched in long time constants and that the timescales in RSC best match the animal’s behavioral strategy. RSC inactivation leads to an impairment in the animal’s ability to use history information to make its decision. Furthermore, our recent studies uncovered that RSC uniquely maintains history information as persistent population activity (*9*, *28*). These identify RSC as a critical cortical area that encodes and maintains behaviorally relevant history information.

When the environment shifts dynamically between periods of stability and volatility, animals need to adapt their behavioral integration timescales (*8*, *24*, *29*–*36*). In theory, RSC neurons could serve as a stable reservoir of heterogeneous history information, and behavioral adaptation could be achieved by flexibly adjusting the readout weights for different neurons by the downstream circuit. Alternatively, individual RSC neurons may alter their time constants of integration according to changes in environmental demands. While RSC itself encodes a wide variety of time constants, RSC may send different temporal information to different downstream areas. For example, the shorter time-constant neurons may project predominantly to the other cortical areas recorded in this study, consistent with the shorter time constants observed in PPC, pM2, ALM, and S1. Longer time-constant neurons may then convey temporal information to other (perhaps subcortical) areas. Cortical pyramidal neurons have been shown to convey distinct information to different cortical and subcortical targets (*37*–*40*). The dynamics of RSC encoding and downstream readout during behavioral adaptations would be an interesting topic of future studies.

Our results resonate with distributional RL (*41*, *42*), which models decision as being made as the combination of parallel estimates of value that vary in their degrees of optimism and learning rates. The advantage of such a system is to simultaneously provide information to the animal about the range of expected outcomes, forming a distribution of prospective reward expectations. Supporting this notion, midbrain dopaminergic neurons represent RPEs with diverse reversal points between positive and negative prediction errors (*41*). As temporal integration may be sped or slowed with larger or smaller learning rates on reward-prediction errors, our model of hyperbolic behavior achieved from diverse speeds of neuronal integration could be consistent with the theoretical model of distributional RL, as either a consequence or cause of the value distribution.

Hyperbolic temporal integration confers a specific behavioral advantage, which is to balance sensitivity to both short-term changes and long-term trends. The distribution of temporal information observed in RSC is capable of producing the hyperbolic-like behavior, and our results suggest a specific cortical substrate and mechanism by which hyperbolic integration might arise.

## MATERIALS AND METHODS

### Experimental design

#### Experimental model and subject details

##### Animals

All procedures were in accordance with protocols approved by the University of California San Diego Institutional Animal Care and Use Committee and the guidelines of the National Institutes of Health. The behavior and neural activity data from two-photon imaging were first reported in (*9*), as was the RSC inactivation data for three of the optogenetic inactivation animals. Behavior from three of the five RSC inactivation animals in (*9*) passed the stricter behavior criteria in this study as described below, and the data were combined with nine additional animals. Other inactivation data are new to this study. Both male and female mice were included in the study because we did not observe sex-related differences in their behavior or neural activity. Mice were originally purchased from the Jackson Laboratory (CaMKIIa-tTA: B6;CBA-Tg(Camk2a-tTA)1Mmay/J [JAX 003010]; tetO-GCaMP6s: B6;DBA-Tg(tetO-GCaMP6s)2Niell/J [JAX 024742]; PV-Cre: B6;129P2-Pvalbtm1(cre)Arbr/J [JAX 008069]; Ai32: B6.Cg-Gt(ROSA) 26Sortm32(CAG-COP4* H134R/EYFP)Hze/J [JAX 024109]). All surgery, behavior training, and experiments were conducted in adult mice (6 weeks or older), on a reversed light cycle (12-hour light/12-hour dark). Mice were water-restricted to ~1 ml/day while undergoing behavior training and experiments.

#### Method details

##### Surgery for calcium imaging and optogenetic inactivation

Animals were prepared for imaging and optogenetic experiments with a large cranial window placed over dorsal cortex, as previously reported in (*9*, *14*). Briefly, mice were anesthetized with 1 to 2% isoflurane during surgery, the dorsal surface of the skull was exposed and cleared of soft tissue with a razor blade and marked with the coordinates of interest. The skull was soaked in saline until the bone became transparent enough to visualize the vasculature patterns on the surface of the brain. We took a photo with both the marked coordinates and vasculature visible and used this as a reference to later identify the cortical area for two-photon imaging and inactivation. A large, hexagonal craniotomy was opened to expose all cortical areas of interest, and a glass window was placed over the surface of the brain. The window was secured to the skull first with a thin application of 3M Vetbond (WPI), then with cyanoacrylate glue and dental acrylic cement (Lang Dental). Last, a custom-machined headbar was attached to the skull, posterior to the window using cyanoacrylate glue and dental cement. Mice were injected subcutaneously with dexamethasone (2 mg/kg) before surgery, as well as buprenorphine (0.1 mg/kg) and Baytril (10 mg/kg) after surgery.

The cortical areas of interest for this study were ALM (1.7 mm lateral and 2.25 mm anterior to bregma), pM2 (0.4 mm lateral and 0.5 mm anterior to bregma), PPC (1.7 mm lateral and 2 mm posterior to bregma), RSC (0.4 mm lateral and 2 mm posterior to bregma), and S1 (1.8 mm lateral and 0.75 mm posterior to bregma) cortex.

##### Behavior task

The dynamic foraging task and training paradigm were described previously (*9*, *28*). In summary, mice were pretrained through a series of behaviors to introduce the task structure and to train to lick to both left and right water delivery ports, using either the BControl system or Bpod to interface with MATLAB and control the behavior apparatus. In the foraging task, head-restrained mice were presented with two lickports monitored with infrared beam detectors. Mice were required to withhold licking during a light-cued ready period (2 to 2.5 s) at the start of each trial, after which the mouse was cued with an auditory tone (10 kHz) to report a choice during the answer period (up to 2 s). After the choice (first lick), they received a feedback tone (left: 5 kHz, right: 15 kHz), and probabilistic water reward. The water volume of the reward was constant at ~2.5 μl per reward. Following reward delivery, a variable-length intertrial interval followed (5 to 7 s), before the ready period marked the beginning of the next trial. Trials in which the mice licked during the cued ready period (“alarm trials”) or trials in which the mouse did not make a choice during the answer period (“miss trials”) were not rewarded and excluded from the analysis.

Reward was assigned to each lickport on every trial according to the reward probability for that lickport in that block. Once a reward was baited to a lickport, it remained available there until chosen. The reward assignment probabilities for the two lickports were either [60%, 10%] or [52.5%, 17.5%]. This probability inverted randomly every 60 to 80 trials with a deterministic order of [60%, 10%], [10%, 60%], [52.5%, 17.5%], [17.5%, 52.5%], [60%, 10%], … with the first block of each session being left high or right high at random.

##### Behavior criteria for session inclusion

For both the imaging and inactivation experiments, mice were trained over weeks for 1 to 2 hours per session per day, to reach “expert” level performance. Each session was evaluated for performance, and only sessions with expert-level performance were included in the analysis, defined as an RL index of at least 0.08, and experience performing the task for at least 15 sessions. The RL index (*9*) quantifies how closely the full RL model [Eqs. 4 to 6] captured the behavior. This was defined as the difference in model fit as follows:

$$\mathrm{RL}\;\mathrm{index}=\sqrt[{n}]{\text{Likelihood of the RL model}}-\sqrt[{n}]{\text{Likelihood explained by bias only model}}$$ (1)

where the bias-only model uses the bias term β_0_ of the full RL model (Eq. 6), and *n* is the number of choice trials in a session. Expert mice usually performed >600 trials in a session.

##### Two-photon calcium imaging and data processing

As previously reported (*9*), imaging experiments were conducted with a two-photon microscope [B-SCOPE, Thorlabs; 16× objective, 0.8 numerical aperture (NA), Nikon] with excitation at 925 nm (Ti-Sapphire laser, Newport), continuously imaged at ~29 Hz. Neurons were recorded from layer 2/3 in a single cortical area and hemisphere per session. We used data for only one population from each hemisphere for each cortical area of a single mouse. The images were processed with a custom-built pipeline (*28*) to correct motion artifacts (*43*) and image distortions (*44*). We then used Suite2p (*45*) to select cells and extract the GCaMP signal, identifying cells first with a user-trained classifier followed by manual inspection. This calcium signal was then deconvolved to obtain estimated spiking activity using a nonnegative deconvolution algorithm (*10*, *11*). This estimated activity for each neuron was *z*-score–normalized across the time series for the entire session before all further analysis. The mean *z*-score activity from the first 2 s of the ready period, when the mouse is withholding licking, was used for the cell activity analysis.

##### Optogenetic inactivation

Cortical inactivation experiments were performed in PV-Cre::Ai32 double transgenic mice via activation of channelrhodopsin-2 in the PV inhibitory neurons. Methods are consistent with (*9*), and this paper includes RSC inactivation from three of the animals from the previous publication. The blue light was directed over the cortical surface through a large cranial window (described above for imaging) with a projector-based light delivery system. Elliptical or circular illumination patterns were produced with Psychtoolbox in MATLAB and projected (DLP projector, Optoma X600 XGA) through a single-lens reflex (SLR) lens (Nikon, 50 mm, f/1.4D, AF) coupled with two achromatic doublets (Thorlabs, AC508-150-A-ML, f = 150 mm; Thorlabs, AC508-075-A-ML, f = 75 mm) to focus illumination patterns over the brain and headbar. A dichroic mirror (Thorlabs, DMLP490L) and a blue filter (Thorlabs, FESH0450) were placed in the light path to pass only blue light (400 to 450 nm).

Cortical inactivation occurred on ~15% of trials, constrained to not be within three trials of the previous inactivation. The light turned on at the beginning of the ready period and turned off with the mouse’s choice or at the end of the answer period, whichever came first. During inactivation trials, the light was directed over the cortical area of interest (one area per session) or over the headbar in control sessions. In the remaining ~85% of trials, the light was directed over the headbar. The light was pulsed at 30 Hz, at an intensity between 2.5 and 6 mW/mm^2^, with a linear ramp down of intensity at offset over 100 ms.

Three inactivation patterns were used: one for RSC, a 2.0 mm × 0.5 mm ellipse, centered at 0.3 mm lateral and 2.0 mm posterior to bregma; one for PPC, a 1.0-mm circle, centered at 1.5 mm lateral and 2.0 mm posterior to bregma; one for pM2, a 1.0-mm circle, centered 0.3 mm lateral and 0.5 mm anterior to bregma. Each pattern was bilaterally symmetric. The control light pattern was directed over the headbar as two 1.0-mm circles centered 1.0 mm apart. The stimulation pattern for each of the cortical and control conditions was light area- and intensity-matched.

Overlapping sets of animals were used for the separate inactivation conditions and between two and four sessions included per animal per condition. RSC: *n* = 10 mice, 26 inactivation sessions; PPC: *n* = 10 mice, 26 inactivation sessions; pM2: *n* = 9 mice, 22 inactivation sessions. Three of the 10 RSC animals and 7 of the RSC inactivation sessions have been previously described (*9*).

#### Analysis details

##### Logistic regression behavioral model

To quantify the strategy of the mouse, we used a logistic regression model to predict the choices the mouse makes in each trial based on the recent experience the mouse has received. The choice on a given trial *t* is predicted by the weighted sum of the rewarded choice (interaction of reward and choice, Rew*C*) and unrewarded choice (interaction of no reward and choice, Unr*C*) in the past 10 trials, along with a constant bias term. The model is

$$\mathrm{logit}[P_{L}(t)]=\sum_{i=1}^{10}\beta_{\mathrm{Rew}C(t-i)}*\mathrm{Rew}C(t-i)+\sum_{i=1}^{10}\beta_{\mathrm{Unr}C(t-i)}*\mathrm{Unr}C(t-i)+\beta_{0}$$ (2)

where *P_L_*(*t*) is the probability of choosing left on trial *t*, Rew*C*(*t* − *i*) is the rewarded choice on past trial *t* − *i* (1 if rewarded on the left, −1 if rewarded on the right, 0 otherwise), Unr*C*(*t* − *i*) is the unrewarded choice on past trial *t* − *i* (1 if unrewarded on the left, −1 if unrewarded on the right, 0 otherwise). β_Rew*C*(*t*−*i*)_ and β_Unr*C*(*t*−*i*)_ are the regression weights for each of the corresponding predictors, and β_0_ is the constant bias term. Model fitting was performed in Python, with the package Scikit-learn (*46*) and the function *LogisticRegression*, solved by gradient descent with the Broyden–Fletcher–Goldfarb–Shanno (BFGS) algorithm.

##### Logistic regression behavioral model for optogenetic analysis

To quantify the effect of inactivation during the ready and answer period on the animal’s choice, we modified the logistic regression analysis to estimate weights separately for the inactivation trials, coded as *Inac*, and control trials, *Ctrl*. Control trials within three trials after inactivation were excluded from the analysis.

Given that inactivation trials occurred on only ~15% of trials in a session, we had a small number of trials for each session relative to the number of regression parameters in the above model. To increase model stability for these experiments, we reduced the number of history trials considered from 10 to 5 trials. In addition, we use lasso regularization with the l1 penalty on both control and inactivation terms, with the hyperparameter selected via cross-validation for each session. Without regularization, the mean CV log-likelihood per trial was −2.23 ± 0.23; with lasso regularization, this increased to −0.64 ± 0.02, a significant increase by two-tailed Wilcoxon signed-rank test with *P* = 7.73 × 10^−14^.

To prevent over-penalization of the inactivation condition, which had fewer trials than the control condition, we subsampled from both sets of trials to have a matched number of trials per condition. Each training set used a random 90% sample of all available inactivation trials and a matched number of randomly sampled control trials. The remaining 10% of inactivation trials and a matched number of held-out control trials were used to evaluate the CV log-likelihood. We iterated this subsample 1000 times with replacement. The reported weights are the mean weights across all iterations for each session.

The resulting model is as follows

$$\begin{array}{rl} \mathrm{logit}[P_{L}(t)]= & \left[\sum_{i=1}^{5}\beta_{\mathrm{Rew}C(t-i)}^{\mathrm{Ctrl}}*\mathrm{Rew}C(t-i\right)\\ & +\sum_{i=1}^{5}\beta_{\mathrm{Unr}C(t-i)}^{\mathrm{Ctrl}}*\mathrm{Unr}C(t-i)+\beta_{0}^{\mathrm{Ctrl}}]*\mathrm{Ctrl}(t)\\ & +\left[\sum_{i=1}^{5}\beta_{\mathrm{Rew}C(t-i)}^{\mathrm{Inac}}*\mathrm{Rew}C(t-i\right)\\ & +\sum_{i=1}^{5}\beta_{\mathrm{Unr}C(t-i)}^{\mathrm{Inac}}*\mathrm{Unr}C(t-i)+\beta_{0}^{\mathrm{Inac}}]*\mathrm{Inac}(t) \end{array}$$ (3)

where the control, Ctrl, and inactivation, Inac, conditions had separate β_Rew*C*(*t*−*i*)_, β_Unr*C*(*t*−*i*)_, and β_0_ regression weights associated with them. The terms Ctrl(*t*) and Inac(*t*) were either 0 or 1, for the corresponding control or inactivation trials.

##### Reinforcement learning behavioral model and simulated behavior

The RL model used to generate simulated behavior was reported in (*9*). This model was modified from the Rescorla-Wagner model to describe mouse behavior in our task, with separately updated action values for the chosen option, *Q*_ch_, and unchosen option, *Q*_unch_

$$\begin{array}{rl} & Q_{\mathrm{ch}}(t+1)\\ & =\left\{ \begin{array}{c} Q_{\mathrm{ch}}(t)+\alpha_{\mathrm{rew}}*[R(t)-Q_{\mathrm{ch}}(t)]\;\mathrm{if}\;\mathrm{rewarded}\;[R(t)=1]\\ Q_{\mathrm{ch}}(t)+\alpha_{\mathrm{unr}}*[R(t)-Q_{\mathrm{ch}}(t)]\;\mathrm{if}\;\mathrm{unrewarded}\;[R(t)=0] \end{array}\right. \end{array}$$ (4)

$$Q_{\mathrm{unch}}(t+1)=(1-\delta )*Q_{\mathrm{unch}}(t)$$ (5)

where α_rew_ and α_unr_ are the independent learning rates for rewarded and unrewarded trials, respectively, and δ is the forgetting rate for the unchosen option. Reward on trial *t* is *R*(*t*) (1 for rewarded, 0 for unrewarded), and the difference between *R*(*t*) and *Q*_ch_ corresponds to RPE. The learning and forgetting rates were constrained to be between 0 and 1. Given the action value for left and right options, which are updated independently, the probability of choosing the left side is

$$P_{L}(t)=\frac{1}{1+e^{-\beta_{\text{Δ}Q}[\beta_{0}+Q_{L}(t)-Q_{R}(t)]}}$$ (6)

where *Q_L_* is the value for the left side, *Q_R_* is the value for the right side, β_0_ is the constant bias term, and β_∆*Q*_ is the sensitivity to the value difference ∆*Q*. This model was fit to the choice patterns of 74 sessions of expert mouse behavior in Python using SciPy (*47*) *minimize* function, with search algorithm L-BFGS-B, to perform maximum likelihood estimation.

In an emulation of the task environment, with the same reward contingencies and block structure as the real task, the RL model algorithm was used to generate choices based on the trial-by-trial updating value from Eqs. 4 and 5 and the softmax function (Eq. 6). This generative model took as inputs the fit parameters from each session of expert mouse behavior. The simulated RL agent ran 10,000 trials for each of the 74 parameter sets, producing sequences of choices and outcomes. These choice and outcome patterns were then fit with the logistic regression (Eq. 2) or decay models (Eqs. 7 and 8) in the identical analysis process as real behavior.

##### Exponential and hyperbolic behavioral models

To evaluate how well the mouse behavior is described by exponential or hyperbolic integration, we quantified the behavior using two explicitly defined decay models that assumed either exponential or hyperbolic decay. Past trials were temporally discounted with an exponential or hyperbolic decay, with time constants fit for each session.

The exponential model was defined as

$$\mathrm{logit}[P_{L}(t)]=\beta_{\mathrm{Rew}C}*\sum_{i=1}^{N}\mathrm{Rew}C(t-i)*e^{\frac{1-i}{\tau_{\mathrm{Rew}C}}}+\beta_{0}$$ (7)

where *P_L_*(*t*) is the probability of choosing left on trial *t*, Rew*C*(*t* − *i*) is the rewarded choice on past trial *t* − *i* (1 if rewarded on the left, −1 if rewarded on the right, 0 otherwise). Up to 15 past trials were considered for this model (*N* = 15), unless otherwise noted. β_Rew*C*_ is the linear regression weight on the kernel, β_0_ is the constant bias term, and τ_Rew*C*_ is the time constant of the exponential.

Similarly, the hyperbolic model was defined as

$$\mathrm{logit}[P_{L}(t)]=\beta_{\mathrm{Rew}C}*\sum_{i=1}^{N}\mathrm{Rew}C(t-i)*\frac{1}{1+\frac{i-1}{\tau_{\mathrm{Rew}C}}}+\beta_{0}$$ (8)

The only difference from the exponential model is the form of the decay function, with the time constant τ_Rew*C*_ of the hyperbolic. For both models, τ_Rew*C*_ was constrained to be greater than 0. These models were fit to the choice patterns of 74 sessions of expert mouse behavior in Python using SciPy (*47*) *minimize* function, with search algorithm L-BFGS-B, to perform maximum likelihood estimation.

To compare the performance of each model, each session was divided into 10 equal sets of trials, 9 of which were used to estimate the exponential and hyperbolic models. At each iteration, the log of the likelihood for the held-out trials was compared as loglik_hyp_ − loglik_exp_. This was iterated across each held-out test set, and the difference in log-likelihood was taken as the mean across all iterations, yielding CV log-likelihood, and normalized by the number of trials per test set.

##### Exponential and hyperbolic behavioral models for optogenetic analysis

The exponential and hyperbolic behavioral models were fit to the behavior by selecting only the inactivation trials or control trials that were more than three trials after inactivation, estimating the models separately for the two conditions. All trials matching this criteria were concatenated for each animal. As with the logistic analysis, we subsampled from the sets of control and inactivation trials to have a matched number of both training and test trials for each iteration of the model fit, bootstrapping 30 times with replacement. Each iteration followed the same procedure of 10-fold cross-validation as above for the exponential and hyperbolic models (Eqs. 7 and 8). The reported CV log-likelihood per trial is the mean across all iterations for each animal.

##### Exponential and hyperbolic cell activity models

To identify the neurons modulated by rewarded-choice history, we averaged the neural activity during the first 2 s of the ready period, during which the mouse was withholding licking. Then, we estimated the influence of the most recent rewarded-choice trial as

$$A(t)=\beta_{\mathrm{Rew}C}*\mathrm{Rew}C(t-1)+\beta_{C}C(t)+\beta_{0}$$ (9)

where *A*(*t*) is the neural activity at trial *t*, Rew*C*(*t* − 1) is the rewarded choice on the immediately preceding trial (1 if rewarded on the left, −1 if rewarded on the right, 0 otherwise), and *C*(*t*) is the choice that the animal will make on the current trial (1 if left, −1 if right) to regress out the anticipatory movement-related activity. β_Rew*C*_ is the linear regression weight on the rewarded-choice history, β_C_ is the weight for the upcoming choice, and β_0_ is baseline offset. Neurons with *P* < 0.05 for β_Rew*C*_ by Student’s *t* test were considered modulated by past rewarded choice.

Among these modulated neurons, we then calculated whether they were more exponential or hyperbolic in their history integration, in an analogous method to the above behavior models (Eqs. 7 and 8). Specifically, neural activity was fit by the exponential model

$$A(t)=\beta_{\mathrm{Rew}C}*\sum_{i=1}^{N}\mathrm{Rew}C(t-i)*e^{\frac{1-i}{\tau_{\mathrm{Rew}C}}}+\beta_{C}C(t)+\beta_{0}$$ (10)

and the hyperbolic model

$$A(t)=\beta_{\mathrm{Rew}C}*\sum_{i=1}^{N}\mathrm{Rew}C(t-i)*\frac{1}{1+\frac{i-1}{\tau_{\mathrm{Rew}C}}}+\beta_{C}C(t)+\beta_{0}$$ (11)

where τ_Rew*C*_ was constrained to be between 0 and 100. The performance of the two models was compared for each neuron as the log-likelihood of the 10-fold CV test set, and the difference was taken as loglik_hyp_ − loglik_exp_ for each iteration and normalized by the number of trials in the test set.

The analysis of exponential time constants was performed on cells that were exponentially modulated (*P* value for β_Rew*C*_ was < 0.05; Eq. 10) in both the first half and second half of the session. The model fit was performed independently in the two halves of the session, and thus, no constraint was imposed that either β_Rew*C*_ or τ_Rew*C*_ be consistent between halves. For these cells, the exponential time constant was estimated across the entire session, and also from two nonoverlapping halves of the session. The full session τ_Rew*C*_ was used for all analyses except for Fig. 3 (D and E), where the two independently estimated τ_Rew*C*_ were compared as a metric of the stability of neural encoding and model estimation.

##### Quasi-hyperbolic behavioral model

The quasi-hyperbolic model is defined as a sum of multiple weighted exponential functions to yield a probability of choosing left or right. From the observed distributions of τ_Rew*C*_ of exponential neurons in each cortical area, we randomly drew between 1 and 15 values of τ and fit the weighting on each exponential kernel to best describe the behavior, following the equation

$$\mathrm{logit}[P_{L}(t)]=\sum_{m=1}^{M}\beta_{m}*\sum_{i=1}^{N}\mathrm{Rew}C(t-i)*e^{\frac{1-i}{\tau_{m}}}+\beta_{0}$$ (12)

where *P_L_*(*t*) is the probability of choosing left on trial *t*, Rew*C*(*t* − *i*) is the rewarded choice on past trial *t* − *i* (1 if rewarded on the left, −1 if rewarded on the right, 0 otherwise), *m* is the number of τ drawn from the observed distribution, β*_m_* is the linear coefficient corresponding to the exponential kernel with time constant τ*_m_*, and β_0_ is the constant bias. The number of past trials considered was *N* = 15. Each set of τ was fit to each of the 74 behavior sessions to yield the 10-fold CV log-likelihood per trial, equivalent to the exponential and hyperbolic behavior models (Eqs. 7 and 8).

### Statistical analysis

#### Linear mixed models

Our experiments included different numbers of observations from different animals and sessions. To account for the inter-animal and inter-session variability, we used linear mixed models for statistical analysis of nested data. The models used in the manuscript are as follows

y∼mdl+(0+mdl∣animal)+(1∣animal:session) (13)

where the fixed effect is the model type, exponential or hyperbolic. A random slope is included for each animal, and a random intercept for each session nested by animal. This model was used to compare the CV log-likelihood of real and simulated behavior in Fig. 1G and fig. S1B.

y∼hemisphere+(1∣animal) (14)

where the fixed effect is the hemisphere from which the session was recorded. A random intercept is included for each animal. The difference between areas was further assessed as the interaction between hemisphere and brain region. This model was used to compare the fraction of cells that prefer left rewarded choice in fig. S2A.

y∼mdl+(0+mdl∣session)+(1∣sesion:cell_id) (15)

where the fixed effect is the model type, exponential or hyperbolic. A random slope was included for each session, and a random intercept for each cell nested by session. This model was used to compare the CV log-likelihood for each cell in Fig. 2D

y∼region+(1∣animal) (16)

where the fixed effect is the brain region from which the session was recorded. A random intercept is included for each animal. This model was used to compare the fraction of cells that were significantly modulated by the exponential history model across different areas in Fig. 3B

y∼inac+(0+inac∣animal)+(1∣animal:session) (17)

where the fixed effect is the inactivation condition, 1 or 0. A random slope is included for each animal, and a random intercept for each session nested by animal. This model was used to compare the effect of inactivation on the sum of reward-choice history (Fig. 4, B and C), CV log-likelihood (Fig 4D), and win-stay and lose-switch strategies (Fig. 4E). The difference between areas was further assessed as the interaction between inac and brain region.

### Statistical software and libraries

Linear mixed models were run in R, using the *lmer* function in the lme4 package (*48*) for parametric tests, or aligned rank transform (*49*) for nonparametric tests. The choice of parametric or nonparametric tests was determined according the normality of the data by Lilliefors test. Log-likelihood measurements were assessed with 10-fold cross-validation. The nonparametric test of distribution medians was bootstrapped 100,000 times, with an equal number of samples drawn from each cortical area and animal, with replacement.

All other statistical analysis and data processing were performed in Python with Statsmodels (*50*). Models were fit in Python with Scikit-learn (*46*); SciPy (*47*), Pandas (*51*), Xarray (*52*), and Numpy (*53*) were also used for data handling, and Matplotlib (*54*) was used for data visualization.
