# Supplementary material for: Exponential history integration with diverse temporal scales in retrosplenial cortex supports hyperbolic behavior
Source: Sci Adv. 2023 Nov 29;9(48):eadj4897. doi: 10.1126/sciadv.adj4897 (PMC10686558; doi:10.1126/sciadv.adj4897)
Supplement: Supplementary file 1 — Figs. S1 to S3 [file sciadv.adj4897_sm.pdf]

Science Advances
AAAS

Supplementary Materials for

Exponential history integration with diverse temporal scales in retrosplenial cortex supports hyperbolic behavior

Bethanny P. Danskin *et al.*

Corresponding author: Takaki Komiyama, tkomiyama@ucsd.edu; Mikio Aoi, maoi@ucsd.edu

Sci. Adv. **9**, eadj4897 (2023)
DOI: 10.1126/sciadv.adj4897

This PDF file includes:

Figs. S1 to S3

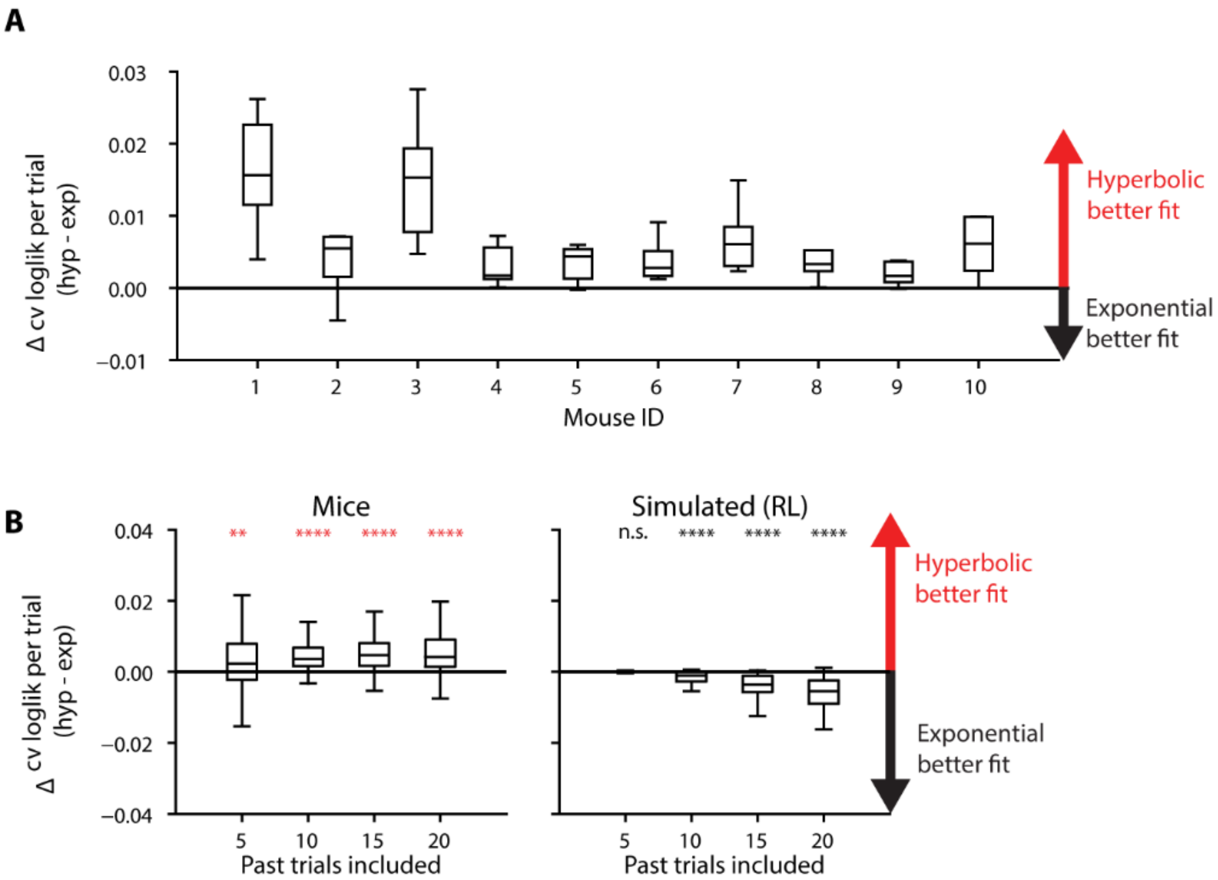

Fig. S1.

Comparison of the model performance, using 10-fold cross-validated loglikelihood normalized by the number of trials, compared between exponential and hyperbolic models across identical train- and test-sets. **(A)** Model-fit comparisons separated by mouse identity for the 10 out of 14 mice with three or more sessions. (**B)** Models estimated with a different numbers of past trials. Box plots at 15 past-trials are reproduced from Fig. 1G. Linear mixed model, n.s $p>0.05$; * $p< 0.05$; ** $p< 0.01$; *** $p< 0.001$; **** $p< 0.0001$.

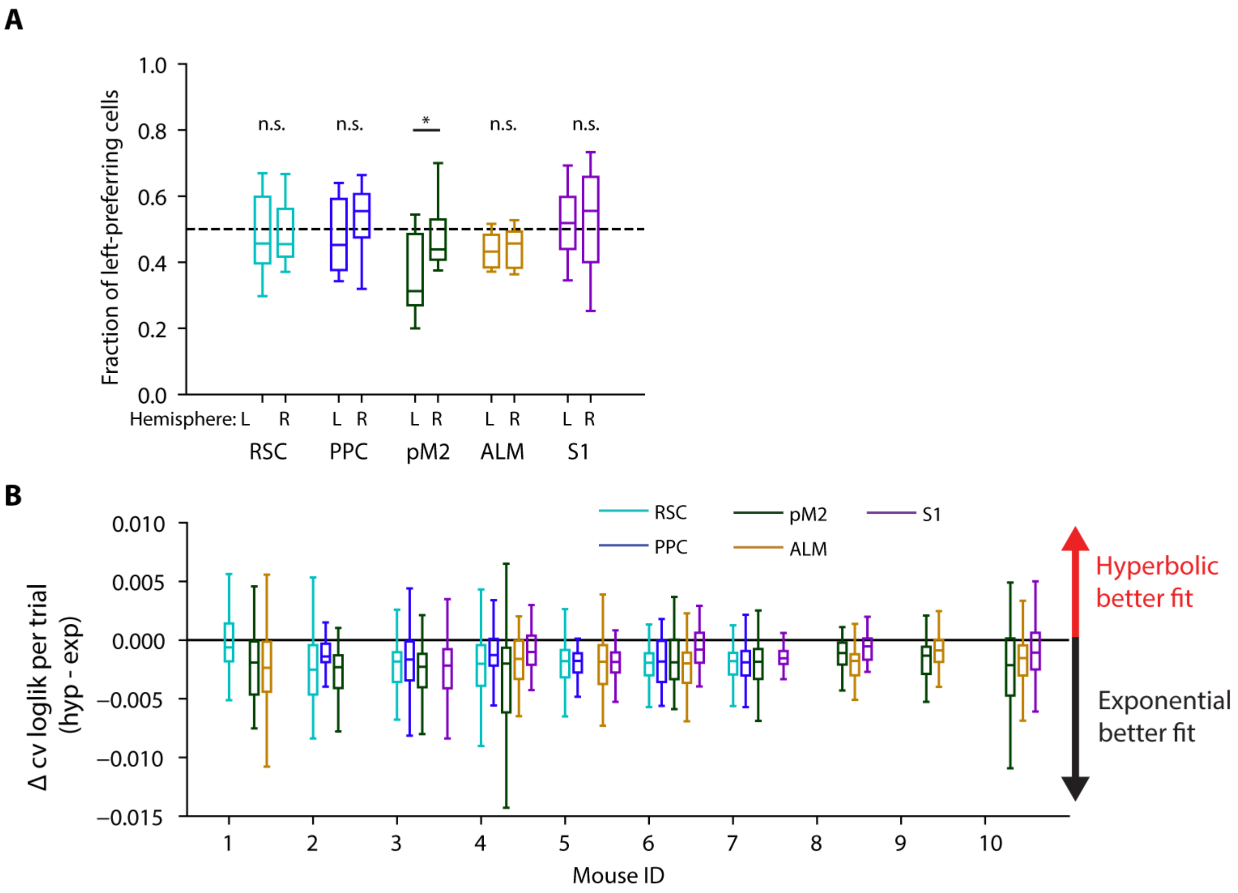

Fig. S2.

(**A**) Fraction of cells significantly modulated by rewarded-choice history with an exponential decay, separated according to the sign of modulation, the brain area, and the hemisphere of the recording session. Linear mixed model, n.s $p>0.05$; * $p< 0.05$. (**B**) Comparison of model performance, using 10-fold cross-validated loglikelihood normalized by the number of trials, between exponential and hyperbolic models across identical train- and test-sets. Separated by mouse identity, but all sessions for each area pooled for each animal. Same animals as Fig. S1A.

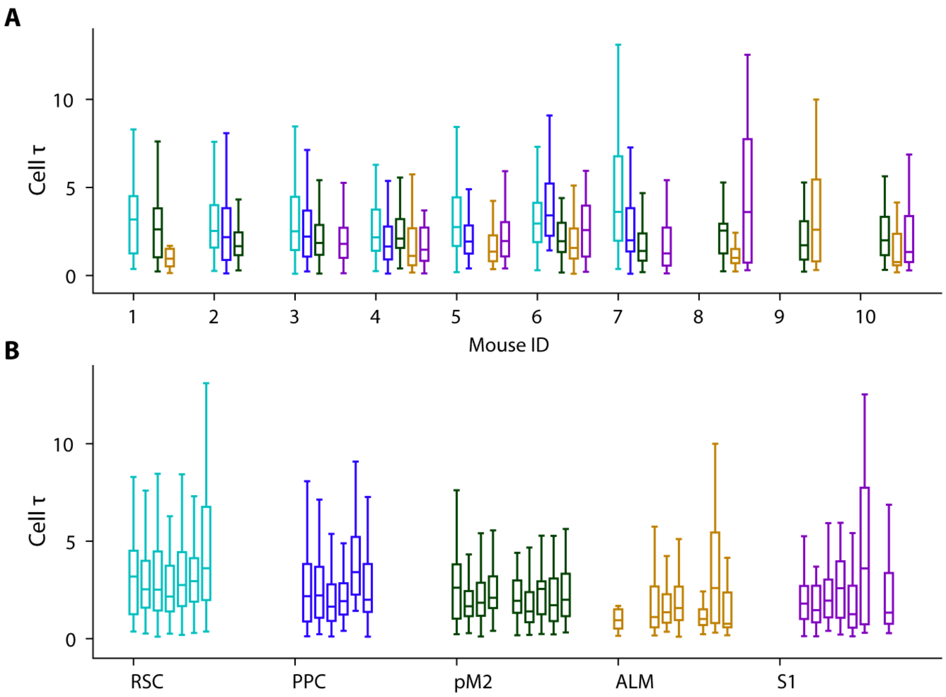

Fig. S3.

(**A**) Distribution of exponential time-constant τ across the significantly-modulated cells in five cortical areas, separated by mouse identity. All sessions for each pooled for each animal. Same animals as Fig. S1A. (**B**) Same data as **A**, sorted by area.
